# Patterns of Internet Use in People Diagnosed With Severe Mental Illness: Qualitative Interview Study

**DOI:** 10.2196/55072

**Published:** 2025-03-28

**Authors:** Ruth Wadman, Lauren Walker, Olivia Taylor, Paul Heron, Elizabeth Newbronner, Panagiotis Spanakis, Suzanne Crosland, Emily Jane Peckham

**Affiliations:** 1 Department of Health Sciences University of York York United Kingdom; 2 School of Medical and Health Sciences Bangor University Wrexham United Kingdom; 3 Department of Sociology University of York York United Kingdom; 4 Department of Psychology University of Crete Rethymno Greece; 5 School of Medical and Health Sciences Bangor University Bangor United Kingdom

**Keywords:** severe mental illness, internet use, qualitative, typology, protective strategies, digital divide

## Abstract

**Background:**

People with severe mental illness (SMI) face profound health inequalities, which may be exacerbated by increased rates of digital exclusion, especially as health services move to online provision. The activities that people carry out online can affect how they feel about the internet and may determine whether a person has a positive or negative experience when using the internet. This, in turn, could affect their mental health. To support people with SMI in using digital technology and the internet safely, it is important to understand the internet and digital technology use of those with SMI and their perceived positive or negative impact on their mental health.

**Objective:**

This study aimed to explore the internet and digital technology use of those with SMI, with particular focus on any association between greater use of the internet and poorer self-reported mental health.

**Methods:**

We carried out a qualitative interview study with 16 people with SMI. The sample was drawn from a wider investigation of the impact of the pandemic and its restrictions on the health and well-being of 367 people with SMI. We purposively sampled from the wider study based on age, gender, frequency of internet use, and self-reported mental health. The data were analyzed by 2 researchers using framework analysis.

**Results:**

Participant experiences fell into 3 broad categories: those who had a positive or neutral internet-based experience, those who had negative or difficult experiences, and low users or those with poor digital literacy. Those who had positive or neutral experiences could be broken down into 2 subcategories: first, those with positive or neutral experiences of the internet who were similar in terms of the activities participated in, feelings reported, and their concerns about the internet, and second, conscious users who were mindful of their interaction with the internet world. Participants with difficult experiences fell into 2 categories: those with worries and fears related to using the internet and those who had difficulty limiting their internet use.

**Conclusions:**

People with SMI, similarly the general population, are expected to conduct more of their activities of daily living online in the postpandemic world. This research shows that most internet users with SMI have positive or neutral experiences. However, our typology reveals subgroups of the population with SMI for whom there is a relationship between internet use and difficult feelings. These subgroups can be identified by asking questions about online activities; time spent online; feelings, difficulties, or issues experienced; and use of gambling, dating, adult content, and conspiracy theory websites. Our findings point to further work in collaboration with people with lived experience to modify and test this typology.

## Introduction

### Background

In the general UK population, changes in internet use and its effects on mental health and well-being occurred during the COVID-19 pandemic. For example, consumption of COVID-19–related news on social media was associated with increased depression and anxiety, but during the pandemic, approximately 50% of UK internet users disclosed going online to support their mental and physical health and to feel less lonely [1,2]. Understanding the experiences of use of the internet in people with mental illness, especially those with severe mental illness (SMI), is important as they may experience unique challenges. People with SMI already experience significant health inequalities compared with people without SMI, including greater levels of physical illness and reduced life expectancy, with an estimated 20-year mortality gap [3,4]. People with SMI are at risk of digital exclusion, which, in the context of increased digital provision of health and other services (eg, video consultations, websites, and apps), could compound these health inequalities.

Research carried out before the COVID-19 pandemic presented a mixed picture of digital engagement and exclusion among people with SMI. Levels of digital exclusion decreased between 2011 (30% digitally excluded) and 2016 (18% digitally excluded) in a longitudinal study of people with psychosis [5]. However, high levels of digital exclusion were observed in people requiring community mental health rehabilitation support (such as residential placements and supported living), where only 14% used the internet [6]. Previous research suggests that most people with SMI have access to a digital device, such as a computer or smartphone. However, low rates of smartphone ownership, particularly in those with psychosis-spectrum disorders, have been reported [7-9].

A qualitative study of digital exclusion in 20 mental health service users (half of whom had psychosis) explored the factors that drive digital exclusion, highlighting a perceived lack of knowledge, being unable to access the necessary technology and services owing to personal circumstances, and the barriers presented by mental health difficulties [10]. Specific facilitators for overcoming digital exclusion included intrinsic motivation and a personalized learning format that reflects the individual’s unique needs and preferences [10]. A qualitative study investigating how service users feel about digital health interventions for people experiencing SMI found that participants reported feelings of empowerment and being liberated because of an increase in choice, tools, and control [11]. The aforementioned study also identified barriers to accessing digital health interventions, with the assumption that these would replace face-to-face care, excluding those with poor access or abilities, and would be problematic for data protection [11].

Previously, people with SMI have reported that they would be less likely to use digital technology when unwell in comparison to when they are feeling well [7]. Research focused on internet use in general and internet use related to mental health for those with psychosis (eg, digital mental health tools, internet-based communities, and discussion boards) has found that general internet use is higher for younger people and those with low symptom severity and fewer cognitive difficulties [12]. Interestingly, use of the internet for mental health information was not associated with symptom severity or cognitive difficulties, indicating that there were no differences in interest in accessing online mental health resources between those with high symptom severity and cognitive difficulties and those with low symptom severity and cognitive difficulties [12].

During the COVID-19 pandemic, we explored the use of the internet, digital skills, and mental health in people with SMI. We found that most (61%) people with SMI were limited or nonusers of the internet despite most (approximately 80%) having access to a digital device and the internet at home [13]. We found that using the internet “a lot” (vs “just a bit” or “not at all”) was associated with younger age, a diagnosis of bipolar disorder compared to a psychosis-spectrum disorder, and a report of a decline in mental health [14]. This finding that high internet use was associated with poorer self-reported mental health was somewhat counterintuitive as we expected greater digital engagement to contribute to greater support and inclusivity. However, we appreciated that it is not only how engaged someone is with the internet but what people do when they are online that affects this. This could determine whether a person has a positive or negative experience using the internet, which could affect their mental health (eg, being online to access support or being online to access potentially scary and stressful information).

In an era in which the use of the internet is becoming increasingly important to access public services, health care, and leisure, it is important to explore the association between greater use of the internet and poorer self-reported mental health to support people with SMI in using digital technology and the internet safely. To do this, we carried out a qualitative study to explore the internet and digital technology use of those with SMI, focusing on time spent online, types of activities, and their perceived positive or negative impact on mental health.

### Objectives

Therefore, the aim of this study was to explore the internet and digital technology use of those with SMI, with a particular focus on any association between greater use of the internet and poorer self-reported mental health.

## Methods

### Participants

Between June 2021 and October 2021, qualitative interviews were undertaken via telephone to explore internet use and abilities for adults who have SMI.

A purposive sample was drawn from the wider Optimising Wellbeing During Self-Isolation (OWLS) study [13]. The OWLS study was a series of 5 surveys tracking the health and well-being of people with SMI during the COVID-19 pandemic. In the initial OWLS study, we asked people whether they would consent to taking part in a qualitative study. From those who consented, we purposively sampled based on age, gender, frequency of internet use, and self-reported mental health. As this study aimed to explore the drivers of the relationship between high internet use and poorer self-reported mental health, the sample was chosen to include individuals with a high use of the internet. In this survey study, we purposively sampled from a group known to use the internet more. This means that the sample reported in this paper had a higher-than-average level of digital skills.

The study team approached 28 participants who had expressed an interest in taking part in an interview study, and 16 (57%) gave informed consent and completed the interviews. After interviewing 16 participants, no new data emerged, and therefore, data saturation was achieved. The final sample included 56% (9/16) female and 44% (7/16) male participants aged between 24 and 68 years; 56% (9/16) of the participants had a schizophrenia-spectrum disorder diagnosis, 25% (4/16) had a diagnosis of bipolar disorder, and 19% (3/16) were diagnosed with another SMI. In total 19% (3/16) of the participants reported outstanding knowledge with regard to use of the internet, 38% (6/16) reported good knowledge, 25% (4/16) reported fair knowledge, and 19% (3/16) reported poor knowledge. Most participants (15/16, 94%) were White British. Most (13/16, 81%) were not employed at the time of the interview. The participants varied in the extent to which they used digital devices and the internet, from limited use to frequent use.

### Interviews

Semistructured interviews were conducted following a topic guide (Multimedia Appendix 1). This was devised from previous findings of the OWLS survey to elaborate on information gathered regarding internet and digital device use in the previous 12 months and the importance of digital skills. The aim of the interviews was to explore how much people were using the internet, what they were using it for, and how using the internet made them feel. This was to further understand the relationship we had previously noted between high internet use and poorer self-reported mental health. Before conducting the interviews, the topic guide was piloted with a member of the OWLS lived-experience advisory group. The interviews were conducted between June 2021 and October 2021 by RW, PH, and LW. The researchers conducting the interviews had either a PhD or Master of Science and were a mix of ages and genders; one of the interviewers has lived experience of SMI. The interviews typically lasted between 30 and 40 minutes.

The topic guide began with introductory questions to gather general information regarding the frequency, duration and main purpose of internet use (eg, “Roughly how many hours a day do you spend doing things online” and “Could you tell me about the different types of activities you do online?”). This was followed by questions to gain an insight into the thoughts and feelings of participants when they used the internet (eg, “Do you think the online activities you do affect your mental health in any way?”). We did not specifically include questions about the use of the internet to receive formal or informal mental health services. This did not preclude participants talking about support via the internet, if they felt it was relevant to their experiences. The interview recordings were transcribed verbatim.

### Analysis

An analysis was conducted with the aim of *exploring the relationship between higher internet use and poorer self-reported mental health.* Following a preliminary analysis by OT, the transcripts were analyzed by LW and EP using framework analysis. This method was chosen as it allows for the identification, description, and interpretation of key patterns in datasets where there is some comparison between groups or concepts to answer a research question. Framework analysis was originally developed for policy research in the 1980s by Richie and Spencer [15] but since then has been used across disciplines (eg, health research and psychology), usually with but not limited to datasets in which there is some comparison between groups or concepts [16,17]:

Framework analysis and applied qualitative research can be a perfect match, in large part because framework analysis was developed for the explicit purpose of analysing qualitative data in applied policy research.

The five stages of framework analysis were followed: (1) data familiarization; (2) framework identification—deductive themes were derived from the research question, interview schedule, and literature and confirmed from data familiarization [18], and these themes were used to create a framework; (3) indexing—researchers (LW and EJP) coded the transcripts to the framework; (4) charting—the data were abstracted and tabulated; and (5) mapping and interpretation—this led to the creation of a typology.

Findings from a 2019 study that looked at internet use in people with mental illness in Poland created clusters of user types (leisure-seeking omnivores, gamers, and passive-selective users) [18]. To create these clusters, they used an adapted version of the Classification of Internet Usage Activities (Table 1) by van Deursen and van Dijk [19]. We used the adaptation by Ng et al [18] as a basis for our classification of what people were doing online, with the further addition of gambling, dating, and adult content websites (from our topic guide), and then divided these activities into consuming (passive internet use), including searching for information and news and shopping, or interaction (active internet use), including social media, study (attending education online), and some types of gaming. This division was made as, from immersion in the transcripts, we speculated that there might be a different experience between those interacting with others online and those with more passive internet use.

**Table 1:** Original classification of activities that the internet may be used for by van Deursen and van Dijk [19] on which Ng et al [18] based their clusters.

Factor	Items
Personal development	Finding online courses and training Following online courses Find vacancies and applying for jobs Independent learning



Leisure	Downloading music and video Hobbies Free surfing


Commercial transaction	Using sites such as eBay Acquiring product information Shopping or ordering products


Social interaction	Using social networking sites Chatting Sharing photos and videos


Information	Using search systems Searching for information

News	News services Newspapers and online magazines

Gaming	Playing games on the internet

### Typology (Mapping and Interpretation)

One of the researchers (LW) created a chart to answer questions about activities participated in online, feelings about the online experience, time spent online each day, and any particular issues or difficulties. A typology was developed from the charting process that categorized participants as having positive or neutral experiences, negative or difficult experiences, and low digital literacy or minimal use. The low digital literacy or minimal use category was classified separately as any feelings or experiences seemed to be in response to apprehension about the unknown or lack of digital skills. As these participants were online for <5 minutes per day, their experiences were not categorized as a response to what they were doing online.

### Ethics Approval

Ethics approval was granted by the Health Research Authority North West – Liverpool Central Research Ethics Committee (reference 20/NW/0276), and all participants gave informed consent before taking part in an interview. Only the researcher who conducted the interview had access to the original recordings, which were securely stored and protected. To ensure anonymity, all participants were assigned unique codes, and personal identifiers were removed during data transcription to safeguard the privacy of the participants. Participants were offered a £20 (US $25.46) voucher as a token of thanks for their time.

## Results

### Overview

Our results originally showed 6 different types of internet users (Table 2). Table 3 shows a simplified version of the typology. Participants with positive and neutral experiences were merged into 1 category as there were few differences between the 2 groups in terms of activities participated in and feelings reported (no particular strong feelings were described), and the 2 groups were similar in terms of their concerns (mainly regarding scam attempts). For the purposes of answering the research question, separating the neutral and positive categories did not provide any additional insights.

**Table 2:** Charting of the activities that people conducted online in terms of frequency of activity and whether carrying out the activity was a positive, negative, or neutral experience.

	Negative or difficult experiences		Positive or neutral experiences			Low use—low digital literacy
	Worries and fears	Difficulties limiting use	Positive	Neutral	Conscious users	
Time daily	Low; average of 45 min	High; average of 9 h, 30 min	Medium; average of 3 h	Low but up from before the pandemic; average of 1 h, 10 min	Medium; average of 4.5 h	Very low; average of <5 min
Activities	Participated in leisure; commercial—retail; information; and gambling, dating, adult content, or conspiracy theory websites Did not participate in social, study, career, news, and gaming	Participated in leisure; commercial—retail; social; career; information; news; gaming; and gambling, dating, adult content, or conspiracy theory websites Did not participate in study	Participated in leisure, commercial—retail, study, and information Did not participate in: career; gaming; and gambling, dating, adult content, or conspiracy theory websites Mixed responses: social	Participated in leisure, commercial—retail, commercial—services, social—video calls and email only, study, and information Did not participate in career; gaming; and gambling, dating, adult content, or conspiracy theory websites	Participated in leisure, commercial—retail, commercial—services, social, study, information, and news Did not participate in gaming; gambling; and dating, adult content, or conspiracy theory websites	Participated in leisure, commercial—services, and social—video calls and email only Did not participate in study; career; information; news; gaming; and gambling, dating, adult content, or conspiracy theory websites
Feelings	Strong negative (fear based)	Negative (boredom and exhaustion based)	Positive	Neutral	Less about feeling and more about choices; both mentioned spiritual belief	Neutral mixed with some distrust and apprehension
Difficulties or issues	Worries and fears about being tracked	Did not necessarily see their high internet use as a problem	Hard to use, scam attempts, or reading bad news	Concerns about online banking and scams	Choosing and being mindful	Lack of understanding or distrust

**Table 3:** Characteristics of the 6 different types of internet users.

	Type of internet-based experience							
	Negative or difficult experiences				Positive or neutral experiences			Low digital literacy or low use
	Worries and fears	Difficulty limiting use	Polarized	Positive or neutral		Conscious users		
Time spent online	Low	High	Moderate	Moderate		Moderate	Very low	
Activities done online	Consuming only	Interacting, consuming, and gaming	Interacting and consuming	Interacting and consuming		Interacting and consuming	Interacting and consuming	
Do these activities include dating, adult content, or conspiracy sites?	Yes	Yes	Yes	No		No	No	
Feelings about the experience with the internet	Strong negative (fear based)	Negative (boredom and exhaustion based)	Mixed	Positive or neutral		Choices over feelings	Neutral feelings about actual use (fear of the unknown)	

The following sections use quotes from the transcripts to illustrate the main findings for each of the original 6 groups and to link the typology to the original data.

### Negative or Difficult Experiences

Participants who had negative or difficult experiences of the internet could be divided into 2 types: those who had worries or fears and those who had difficulties limiting their internet use.

#### Worries and Fears

This was a group of participants with worries and fears about being online who described strong negative (fear based) emotions in relation to their internet use; did a limited number of activities online, which involved mainly consuming content and very little interacting; had used gambling, dating, adult content, or conspiracy theory websites (specifically, conspiracy theory websites were mentioned); and spent an average of 45 minutes per day online. The participant quotes from this group illustrate the participant experiences typical of this group in terms of time spent online; activities participated in; feelings, issues, or difficulties; and experiences with gambling, dating, adult content, or conspiracy theory websites (Textbox 1).

**Participant quotes from the worries and fears group.**

**Time**

- “So, I mean I’ve gone through different periods of using the internet. Sometimes we use if quite intensely over times like now I’m just using it for, like I don’t know, I’m looking at computer programming at the minute. So I just usually look for bits of information about computer programming that I need and things like that and [unclear]. About half an hour a day or summat like that I’d say.” [Participant 274]
- “My general use no more than, definitely no more than an hour.” [Participant 167]

**Activities**

- “Amazon, I’ve used Amazon to get a few pairs of tracksuit bottoms and things like that...Yeah, I actually do quite a bit of shopping on-line to be fair. I get quite a lot of books on the internet.” [Participant 274]
- “Yeah just food shop and sometimes a little bit of, you know, search words or shops and that on Google search and that but pretty much just shopping and that’s it. Not very often like or I might check my bank on-line, you know, things like that or I pay a bill or summat.” [Participant 167]
- “YouTube is a big one. I don’t know much about Netflix. My brother bought me an Android tablet and put Netflix on it but I can’t remember what’s happened with that though.” [Participant 274]

**Feelings**

- “I don’t know the best way to describe how it makes me feel. It doesn’t make me feel good I know that much.” [Participant 167]
- “Yeah, well it (the internet) has and it hasn’t (had a positive impact on my mental health) because I’ve tried to kill me self and finished up in hospital.” [Participant 274]

**Issues or difficulties**

- “I recently went on Facebook for probably the second time I’ve been on Facebook. What I don’t like about Facebook is that you set up an account and then you can’t delete it, so in a way it’s a bit...there’s no-one to look after you on Facebook. I mean you can report stuff that you think is not there to be put up on-line, you can report things and stuff like that. There’s no-one for you to contact if you want to delete your account. You can’t get rid of it and stuff like that. Plus, I think to be fair it’s a bit spooky because I don’t know if police can pull you for certain things if you’ve been doing something and you’ve mentioned it all on Facebook.” [Participant 274]

**Gambling, dating, adult content, or conspiracy theory websites**

- “Yeah, I used to do a bit of [unclear] years ago, you know, not very often but, you know, here and there like but that’s not summat I’ve done in over 4 years.” [Participant 167]
- “Well I’ve never really been a gambler if I’m being honest but other things, you know.” [Participant 167]

#### Difficulties Limiting Internet Use

This was a group of participants who had difficulties limiting their internet use who described negative feelings regarding boredom and exhaustion in relation to their internet use; did many different things online (this was the only group that reported gaming); used gambling, dating, adult content, or conspiracy theory websites (specifically, gambling and dating sites); and spent an average of 9 and a half hours per day online. The participant quotes from this group illustrate the participant experiences typical of this group in terms of time spent online; activities participated in; feelings, issues, or difficulties; and experiences with gambling, dating, adult content, or conspiracy theory websites (Textbox 2).

**Participant quotes from the difficulties limiting internet use group.**

**Time**

- “Most of them [hours] when I’m not asleep. A lot of hours, most of the day I use the internet...I work from home so I’m on a laptop from about 8 till 4 or 9 to 5. I game a lot in the evening, use the night on-line services. TV is on a lot. Always on my phone, so constantly.” [Participant 216]

**Activities**

- “I do tend to look at quite a lot of stuff and buy quite a lot of stuff which I probably don’t need. I think the tablets make me do that as well [The medication that I take].” [Participant 215]
- “My screen time on my phone is quite high, so I’ll scroll through Twitter throughout the day, that’s where I get most of my news. If something big has happened that’s where I’ll usually see it first. Tik Tok is really bad for a rabbit hole so I just keep scrolling because it’s addictive. Facebook chatting with my friends. Instagram. Yeah all of my banking is on my phone. Pretty much everything I do is on my phone, so I use contactless payments, I don’t even use cards, like physical cards. Let me just look at the apps I have. Uber, so I use that like all the time. I haven’t got that much public transport. I have an electric toothbrush that has an app. My toothbrush is connected to the internet.” [Participant 216]

**Feelings**

- “Well I think it’s due to my isolation and the tablets that I take, I’m sort of bored, so I’ll try and buy something, electrical goods or something or something else that I don’t need, normally anyway. Yeah, that’s quite a big part of my internet usage. I’m trying to cut down on that. But yeah, it’s quite interesting. It’s quite engaging.” [Participant 215]
- “Well yeah it means that I’m glued to a screen all day and there’s no like face to face meetings to communicate with people, it’s really different and it seems to be more exhausting and I Googled why, it’s because you can’t interpret people’s body language or like the only communication you’ve really got is speech and that’s quite exhausting trying to work out things about other people.” [Participant 216]

**Difficulties or Issues**

- “It makes me want to buy stuff more. I would say to sort of fill any other void in yourself, I’ll buy something I’ll treat myself to something. It’s all the time. I’ve realised that I do it quite a lot.” [Participant 215]
- “I find it hard to go outside now because I’m so used to being in and talking to people online.” [Participant 216]
- “So like I’ll scroll through my phone in bed a lot, it’s harder to switch off and because obviously blue light from screens that I’m looking at all the time, it’s harder to go to sleep and even...because we don’t have an aerial in our house. We just use like YouTube and stuff, so literally everything we do is, it’s not even like going through into the TV, it’s just the internet all the time. If the internet goes down then my life is ruined.” [Participant 216]

**Gambling, dating, adult content, or conspiracy theory websites**

- “I get addicted to things quite easily. So, if I think something is unhealthy, like mentally, I probably [unclear]. I’ve gambled a little bit.” [Participant 216]
- “Not for a while anyway, not since I was younger, so yeah. Yeah, I don’t really use any of that stuff to be honest. I’ve tried internet dating once or twice but that never works. That’s about it really.” [Participant 215]

### Positive or Neutral Experiences

#### Overview

Those who had positive or neutral experiences were categorized into 3 groups: those with positive experiences of being online, those describing neutral experiences, and conscious users. The positive and neutral experience groups were merged into 1 category as the 2 groups did not appear to be significantly different from each other.

Those with positive and neutral experiences were a group of participants who described positive or neutral feelings in relation to their internet use; did a mixture of things online involving both interacting and consuming; did not use gambling, dating, adult content, or conspiracy theory websites; and spent 1 to 3 hours per day online. The participant quotes from this group illustrate the participant experiences typical of this group in terms of time spent online; activities participated in; feelings, issues, or difficulties; and experiences with gambling, dating, adult content, or conspiracy theory websites (Textbox 3).

**Participant quotes from the group with positive and neutral experiences.**

**Positive**

Time:

- “I suppose then these days when I get up and look at my phone, I will go through WhatsApp and I will look at emails and, you know, sometimes there’s the odd message from somebody that doesn’t use WhatsApp. But I’d say generally [unclear 28.20] spend an hour or so messing about on it. To me it’s a lot, remember people of my age didn’t get used to computers at school and all that, you know, we are the older generation.” [Participant 533]
- “Not much at all. Two hours.” [Participant 182]

Activities:

- “Like I say I’ve got my bank account and mobile banking on my phone. I use all that which is handy as hell. I use it all the time. I’m forever doing that. I transfer money.” [Participant 348]
- “Googling stuff about me own mental health...I like to read up on that...It was giving us more information about what was actually going on in me head.” [Participant 183]
- “It’s mainly social media, Twitter, Instagram, Facebook, catching up with family and friends really and messaging.” [Participant 182]

Feelings:

- “I wouldn’t say it’s [internet activities] have any negative effects on us, no.” [Participant 183]
- “Yeah I would say it’s helped us [having the internet]. Just having that extra thing there. Just like keep your mind ticking over, you know, help.” [Participant 183]
- “Oh yeah very pleasurable especially with the jokes and knowing that some people enjoyed some jokes and it’s a laugh and then they send you a little smiley face back. I think that’s all very positive especially in the pandemic. It has for me become the normal way to contact people.” [Participant 533]
- “I suppose I wanted to feel part of the world if you know what I mean, part of like by going on twitter and Facebook, it felt like I was participating.” [Participant 182]
- “Music tends to chill me out and calm me down, so I just listen to that over again. I mean YouTube, all your songs are there for you and it’s free too.” [Participant 183]

Difficulties or issues:

- “I wish they were easier to use sometimes, some of these websites...They don’t always have instructions as you’re going along, you have to find out what to do yourself or they say press there and it comes up with something else. Some of them are just quite difficult to use.” [Participant 182]
- “I’ve given up watching the news years and years ago because being bipolar you just don’t want to listen to the news because it’s all bad news. So it’s just that really. You get your bad news through the internet now, not necessarily listening to the news on TV or reading newspapers but that’s just the way things are, isn’t it.” [Participant 533]
- “I’ve had a few cranks that have come over the internet and tried to get money out of me but, you know, I’ve just got rid of them, so that’s happened. And I’ve had a few people try to extort money out of me over the internet which I didn’t fall for. I was just laughing at them. I knew what they were doing, so I just played along with it and then when they realised that I wasn’t going to pay out any money they soon got rid of me.” [Participant 348]

Gambling, dating, adult content, or conspiracy theory websites:

- “So, I wouldn’t say that...the negative content, you know, it is an issue, I just don’t bother with it. I don’t read it. I read hardly anything. I don’t get in contact with stuff that stresses me apart from the adverts.” [Participant 533]
- “Well what I did was I got this bloody, it was [name] next door and it was one of these dating apps, you know these dating app things. I just wanted rid of it. But I got it and I thought this is absolutely useless. I don’t know why I went on and it took me ages to get rid of it. I didn’t like it. I just want to meet people naturally rather than going on-line and doing it.” [Participant 348]

**Neutral**

Time:

- “Not a lot to tell you the truth. Oh, I’d say about 20minutes, if that.” [Participant 314]
- “Oh roughly? Probably about an hour.” [Participant 332]

Activities:

- “So, we have a club in our village, the supper club which meets once a month and obviously we weren’t able to meet, so they did run some Zoom sessions on that. Yeah that was a good social thing.” [Participant 317]
- “As I say my husband tends to use it more than I do. I do try learn off him, you know, what he’s doing and stuff and that’s it really.” [Participant 314]
- “Well I’ve been doing shopping. What else? Looking up holidays and things like that...Oh I’ve been on Zoom talking to people [family and friends]...I was playing scrabble at one time and doing jigsaws on-line as well.” [Participant 332]
- “I don’t do the social media stuff. I don’t get involved in it at all.” [Participant 317]

Feelings:

- “...yes, it [online scrabble and jigsaws] helps me sort of unwind a bit.” [Participant 332]
- “I’m still a bit apprehensive about it [online banking] because I’m frightened that we’re going to get conned [laugh].” [Participant 314]
- “Yeah I would probably say that, yeah it [use of the internet] probably wasn’t a bad thing when we were in lockdown.” [Participant 317]

Difficulties or issues:

- “I’m frightened, you know, that something will happen and we’ll lose money. But I mean, touch wood, since we’ve been doing it we seem to be doing it right and we’ve had no problems with it.” [Participant 314]

Gambling, dating, adult content, or conspiracy theory websites:

- “No, I don’t gamble. I don’t go on the adult sites or anything like that. In fact, I wouldn’t know how to get on them I don’t think, so yeah...No I don’t do anything like that.” [Participant 314]

#### Conscious Users

This was a group of participants who used the internet consciously who talked more about choices over online activity than feelings; used the internet for many things (with the exception of gaming); may have come across gambling, dating, adult content, or conspiracy theory websites and chose not to use them; and spent an average of 4 and a half hours per day online. The participant quotes from this group illustrate the participant experiences typical of this group in terms of time spent online; activities participated in; feelings, issues, or difficulties; and experiences with gambling, dating, adult content, or conspiracy theory websites (Textbox 4).

**Participant quotes from the group of conscious users.**

**Time**

- “So probably about 3 to 4 hours at work and maybe 2 hours at home.” [Participant 178]
- “I don’t know. It fluctuates. It would be, you know, going on...I don’t actually have a laptop. I either go on the phone or we have an iPad.” [Participant 352]

**Activities**

- “So, you know, even during that time I’ll be mostly using systems, you know, using Chrome, using different systems for work. Maybe have some music while I’m working.” [Participant 352]
- “Yeah, I mean Facebook, Amazon. Yeah, you know, I’ve just acquired a kindle, so yeah. Facebook, Outlook, WhatsApp, Amazon, I’ve got Prime which is a blessing! You know maybe reading or listening to something like a Jewish Torah lecture or something like this. Lesser organise Instagram. I’ve not done this in a while but I was actually using Zoom so in addition to these like, you know, communal Zoom calls that I mentioned like courses that I do, I was using Zoom for some remote one to one Alexander technique lessons.” [Participant 352]
- “Yeah, so at work I do a lot of academic research, reviewing papers and stuff and ordering, stuff like that. At home it tends to be kind of messaging, social media based kind of stuff.” [Participant 178]
- “I use banking like once a week to make sure everything looks fine and sometimes we order online but only kind of a necessity really.” [Participant 178]

**Feelings**

- “Yeah I’m definitely not negative. Just probably being mindful, you know, for example, Amazon not to spend too much. To get unnecessary items.” [Participant 352]
- “I think maybe also realising how much time I was spending on it because I’m now a lot more conscious about how much I am spending on the internet and a lot of the time not being very productive and I’ve realised I would rather be doing other things. I think it was helpful in different ways at times but there was definitely a point where it was more harm than good because a lot of the time scrolling social media was just like doom and gloom and it was really hard not to focus on it.” [Participant 178]

**Difficulties or issues**

- “...keep in touch with people and that became quite difficult and I had to make some self-preservation decisions. I don’t need to be up-to-date with how my friends are right now so maybe stepping away from that.” [Participant 178]
- “Okay. I mean yeah, I mean yeah what to say. I mean pornography. I mean I think, you know, our society, you know, pornography...look before you even get to hard core stuff that’s on-line, you know, on the various sites but I mean just the objectification pictures and kind of stuff. I mean you don’t even have to go on sites on the internet to see that, that’s on mainstream tv shows now, you know, or, you know. I mean just not even looking for it, I mean this is what people post really. I mean people are posting all kinds of stuff on Instagram, Facebook. Yeah even before you get to like all the kinds of specific like hard core stuff you can get into on the internet. I mean there’s plenty more you can get on, it’s just out there. I mean Netflix or whatever, you know.” [Participant 352]

**Gambling, dating, adult content, or conspiracy theory websites**

- “...very easily accessible, objectification images there when not looking for them.” [Participant 352]
- “I don’t really use any of them. I don’t gamble or anything like that because I would lose all my money, so yeah. I don’t think I have much to contribute to that one.” [Participant 178]

### Low Digital Literacy or Low Use

This was a group of participants with low digital literacy and minimal use who described neutral feelings about their actual internet use and expressed distrust with regard to potential use. This group described using the internet for leisure, services, and emails or video calls only and mentioned others in the household doing internet-based tasks on their behalf; did not use gambling, dating, adult content, or conspiracy theory websites; and typically spent 0 to 5 minutes a day online. The participant quotes from this group illustrate the participant experiences typical of this group in terms of time spent online; activities participated in; feelings, issues, or difficulties; and experiences with gambling, dating, adult content, or conspiracy theory websites (Textbox 5).

**Participant quotes from the group with low digital literacy or low use.**

**Time**

- “Oh, very little. I might get on it once a month.” [Participant 143]
- “Not hours but minutes [per day].” [Participant 159]

**Activities**

- “I only do sort of like go on YouTube and I don’t really do anything else.” [Participant 159]
- “I do emails. Hmm what else? Finding things out and that’s about it.” [Participant 143]

**Feelings**

- “I’m still a bit apprehensive about it because I am frightened that we’re going to get conned...I’m frightened, you know, that something will happen and we’ll lose money.” [Participant 143]
- “I’ve never bought anything on-line because I basically don’t trust with my card details on the internet. I’d rather go to a shop.” [Participant 159]
- “I’m very aware of problems with the computer, so I’m always a bit careful what I’m doing and when. I don’t particularly trust them. I’ve always been like that.” [Participant 143]

**Difficulties or issues**

- “Well if you’re trying to find something out, you can’t get to the right page or you don’t seem to have the information to hand and I find it takes me ages to do anything.” [Participant 143]
- “...tried Facebook but couldn’t work out how to use it and tried to read the instructions for Facebook.” [Participant 159]

**Gambling, dating, adult content, or conspiracy theory websites**

- “I wouldn’t know how to do any of that at all or do that myself, no.” [Participant 159]

## Discussion

### Principal Findings

The patterns of internet use and feelings in people with SMI described in the typology are of interest because, as more of an online presence is necessary for many life tasks and situations, including access to health services, it is important to understand the barriers to inclusion that specific populations may face [20]. The typology of internet users with SMI demonstrates a breadth of experiences and challenges when using the internet. When thinking about addressing the digital divide in this population, a generic approach to digital upskilling (eg, a self-navigated online course) is unlikely to be sufficient.

In response to the findings of the OWLS survey and qualitative work that highlighted a lack of digital skills as the driver of the digital divide in the population with SMI, there may be moves to support people with SMI in increasing their digital skills [13,14]. The experiences of specific subsections of this potentially vulnerable population need to be understood and considered to provide tailored support that understands population-specific barriers and use patterns.

The typology developed in this work revealed patterns that deserve further investigation and offers some guidance at this stage for potential subsections of the population with SMI for whom being online could be problematic.

The *worries and fears* group seemed to try to limit their internet use to mainly consumption activities. Their descriptions of their experiences and feelings suggest that interacting online could be problematic for their mental health. The group who struggled to limit their internet use did a wide range of activities online (including consuming and interacting activities), said that they might seriously struggle without the internet, and talked about shopping in response to isolation and boredom.

Interestingly, both groups of participants who described negative or difficult experiences described using or having previously used gambling, dating, adult content, or conspiracy theory websites, whereas those with positive or neutral experiences or low digital literacy did not report internet use of this type. The exception to this was the conscious use group, in which a participant described this kind of content being difficult to avoid but choosing not to engage with it.

Conscious use is of interest in that these participants described a specific type of relationship to their internet use where they were aware of the possible downsides and negative impacts of the web and made active choices to mitigate these effects. Perhaps there is some learning from the experiences of the conscious user group that could be applied to the group with negative or difficult experiences. This could include the option of conscious limitation of internet use to consumption activities (rather than interaction) for people whose experiences most closely align with the *worries and fears* group; the option of mindfulness about time spent online and types of activities for the group who find it difficult to limit their use; and potential co-designed education on ways to handle internet-based content such as gambling, dating, adult content, and conspiracy theory websites. Perhaps any attempts to increase presence online for this population could include a mindful internet use session created in collaboration with people with SMI.

These postulations about the potential application of the insights from this interview study suggest a very strong case for collaborative work with people with lived experience of SMI. This typology could be shared for validation or otherwise from a lived-experience perspective, and such input could be used to modify and refine the typology. Following this, any advice on internet use for subsections of the population with SMI should be created and verified from a lived-experience perspective (co-design) and eventually tested in a larger, probably quantitative study.

From the standpoint of the results of this work so far, services that support people with SMI should be mindful and understanding of their concerns and barriers faced in relation to using the internet and doing online activities. People with SMI should be offered a one-to-one conversation before embarking on digital upskilling to identify any concerns that may require tailored support.

Any digital upskilling endeavors with people with SMI should devote time to informing them how to safely use the internet and also reassuring them that it is possible to keep themselves safe online.

### Limitations and Strengths

Although this research was carried out during the COVID-19 pandemic, we believe that many of the difficulties described by the participants, such as fears about internet scams, are applicable to the postpandemic world [21,22]. The *worries and fears* participants described little or no change in their internet use as compared to before the pandemic in terms of time spent online. Changes to services and activities demand a greater web presence, particularly access to health services [20]. Corresponding author LW has direct lived experience of SMI and brings this perspective to the work. LW can only bring their own perspective to this work, and their background in coproduced research leads them to believe that further collaborative and coproduced work is required in this area.

### Comparison With Prior Work

A 2015 systematic review showed that people with psychosis seem to spend more time in chat rooms or playing online games than control groups. The use of other internet-based tools such as Facebook or communication through email was lower or the same as that of controls. Online social networking was used by people with psychosis for establishing new relationships, maintaining relationships or reconnecting with people, and receiving online peer support [23]. Our findings suggest that a subgroup of people with SMI may use the internet in these ways and have trouble limiting their use.

Previous research conducted internationally [24] reveals a similar level of internet use in inpatients with schizophrenia to that of the general population and a difference in terms of higher use of the internet as a source of health information than in the general population. Patterns and levels of use may vary depending on global location and inpatient or community settings.

As far as the authors are aware, there has been little research specifically looking at the link between greater internet use and poorer self-reported mental health in people with SMI. This work builds on a previous body of work rather than acting as a comparator with previous findings.

This typology builds on our series of previous publications [13,14,25] investigating internet use, digital health literacy, and the digital divide in people with SMI. We know from previous quantitative research that people with SMI are more likely to be limited users or nonusers of the internet [13] and, as a population, are lacking digital skills [14]. For some of the participants in this study, the internet was seen as useful for practical tasks such as internet shopping and banking. It was important for staying connected and accessing health information. Participants did not always speak directly about internet use affecting their mental health. Worries, fears, and difficulty limiting internet use, as well as feelings of distrust and apprehension about using the internet, were notable, and a subsection of participants appeared to be having negative experiences in relation to going online.

In a previous study, people with SMI were found to increase their use of the internet over the course of the pandemic, but approximately half rated their knowledge of the internet as fair or worse than fair [14]. In addition, this group was found to have moderate levels of digital health literacy. This underpins the need for digital skill training for people with SMI who would like to access this, with a focus on digital health literacy (eg, the ability to find and understand health-related information online and apply this knowledge to make health care decisions and self-manage their conditions). A partnership approach with a clinician for seeking mental health information has been found to be useful for people with psychosis, and this should also be considered [26].

### Conclusions

This was a small sample, and our findings require further investigation. They are not generalizable. However, as far as we know, there is little research looking at patterns of internet use and feelings in this population, and the authors believe that the findings and typology can provide the beginnings of guidance for tailored support for people with SMI who wish to increase their digital skills or presence on the internet.

Our research shows that most internet users with SMI have positive or neutral experiences. However, our typology reveals subgroups of the population with SMI for whom there is a relationship between internet use and difficult feelings. These subgroups can be identified by asking questions about online activities; time spent online; feelings, difficulties, or issues experienced; and use of gambling, dating, adult content, and conspiracy theory websites. This exploration is particularly pertinent in light of findings highlighting the need for training and support among people with SMI to increase digital skills, facilitate digital engagement, and reduce digital exclusion [25]. Our conclusions point to further work in collaboration with people with lived experience to modify and test this typology.

## Appendix

Multimedia Appendix 1 Topic guide.

## Abbreviations

OWLS: Optimising Wellbeing During Self-Isolation
SMI: severe mental illness

## Data Availability

The datasets generated or analyzed during this study are available from the corresponding author on reasonable request.
