# Supplementary material for: Patterns of Internet Use in People Diagnosed With Severe Mental Illness: Qualitative Interview Study
Source: J Med Internet Res. 2025 Mar 28;27:e55072. doi: 10.2196/55072 (PMC11992490; doi:10.2196/55072)
Supplement: Multimedia Appendix 1 [file jmir_v27i1e55072_app1.docx]

**OWLS Digital Study**

**Topic Guide for Qualitative Interviews**

**Introductory Notes:**

- Thank the participant for helping us with the OWLS digital study
- If necessary, briefly remind them of what the study is about…
- Explain that the interview is likely to take 30 to 40 minutes and check that it is still a good time to talk to them
- Explain that we need to confirm their consent to take part in the interview
- Turn on the recorder
- Go through consent form
- Turn off the recorder
- Check if they have any questions or concerns before starting the interview

*** TURN THE RECORDER AGAIN ***

1.Roughly how many hours a day do you spend doing things online? Is that mostly for work or non-work-related activities?

2. Could you tell me about the different types of activities you do online (i.e. using the internet)? [cover work and personal]

Explore: daily living tasks like shopping, banking, booking appointments; following social media (e.g. Facebook, Instagram); communicating with friends/family/work colleagues (e.g. Zoom, Skype, WhatsApp video calls); entertainment/information seeking (e.g. YouTube, internet searches); online computer games; getting information about mental health/medications. [**make a note of the activities mentioned*]

3. Since the pandemic started, has there been any change in the activities you do online? (e.g. talking to family on video chat or starting online shopping or online work activities)

If yes, explore the reason for the change(s).

4. Again, since the pandemic started, has there been any change in:

a) The amount of time you spend doing online activities?

If yes, explore the reasons for the change and whether they are for practical reasons (e.g. working from home) or for well-being (e.g. feeling lonely) or for other reasons.

b) The time of day you do your online activities?

If yes, explore the nature of the change and whether they see it as ‘good’ or ‘bad’ (e.g. deciding to switch off from social media at a set time in the evening or feeling they have to look at work email outside working hours)

5. Thinking about the activities you do online, could you tell me a little about how [each of them?] make you feel when you do them? [**refer to activities mentioned in question 3*]

Explore:

a) neutral (e.g. ordering my shopping); supported/happy/positive (e.g. talking to my family/friends); worried/sad/negative (e.g. disconnected, missing people)

b) whether they have ever had a particularly helpful/good experience online

c) whether they have ever had a particularly unhelpful/bad experience online

6. Do you feel your mental health has been affected by the pandemic, either positively or negatively?

If yes, briefly explore why.

7. Do you think the online activities you do affect your mental health in any way? [cover both work and personal]

Explore:

a) Any direct impact on or link between online activities and their mental health symptoms (e.g. voices, hallucinations, paranoid ideas) and/or;

b) Impact of different types of activities and the possible reasons for the impact and/or;

c) Any strategies they adopted to manage the impact.

8) Sometimes people do online activities that they may not feel comfortable talking about in public (e.g. online gambling, dating habits, adult content, conspiracy theory websites). We are interested in hearing about these experiences and how they may relate to mental health and wellbeing. Is there anything you would like us to know about this?

9. Is there anything else you’d like to tell me about your experience of online activities or your use of the internet before or during the pandemic?

a) Have there been any big changes in your life since the pandemic started back in March 2020, that have affected your online activities or use of the internet?

Explore: personal/living circumstances; employment/voluntary work; finances; other significant changes

**Closing Notes:**

- Thanks the participant for taking part in the interview
- Check if they have any final questions or concerns
- Add a friendly question/comment e.g. what have they have planned for the rest of the day or enjoying/avoiding the weather etc.
